# Isolated Congenital Hyponychia and Anonychia in a Neonate: A Rare Case

**DOI:** 10.7759/cureus.102176

**Published:** 2026-01-23

**Authors:** Sanjaykumar Tanti, Sandeep Jhajra, Veera Harshini, Anil Kumar

**Affiliations:** 1 Department of Pediatrics, Manipal Tata Medical College, Manipal Academy of Higher Education India, Jamshedpur, IND; 2 Department of Pediatric Medicine/Neonatology, Manipal Tata Medical College, Jamshedpur, IND

**Keywords:** anonychia, congenital, isolated, nail disorders, nail hypoplasia, neonate

## Abstract

Congenital hyponychia and anonychia are extremely rare nail disorders characterized by the partial or complete absence of nails. These conditions may be inherited or associated with in utero exposure to teratogenic substances. A full-term male neonate underwent routine head-to-toe examination in the labor room. The baby was noted to have both incomplete (hyponychia) and complete (anonychia) absence of fingernails and toenails. No other systemic abnormalities were identified. Isolated congenital hyponychia and anonychia are uncommon findings in neonates. In the absence of syndromic features, the condition generally carries an excellent prognosis.

## Introduction

Complete absence of the nails at birth is referred to as congenital anonychia [1]. Congenital nail anomalies are uncommon and may present as either isolated conditions or part of broader genetic syndromes. Nail hypoplasia (partial absence of nails) and anonychia (complete absence) are especially rare, with only 74 cases reported worldwide till 2017 [2,3]. Anonychia may present as an isolated anomaly or as a feature of various syndromic conditions. It has been reported in association with syndromes such as nail-patella syndrome, anonychia accompanied by hypoplasia or dysplasia of the distal phalanges, microcephaly, brachydactyly (as seen in Cook’s syndrome), Zimmermann-Laband syndrome, and DOOR syndrome (characterized by deafness, onychodystrophy, osteodystrophy, and intellectual disability) [4-6]. When they occur in isolation, without systemic involvement, they are typically benign and carry a good prognosis. These disorders may be inherited or arise sporadically due to mutations affecting nail development pathways or to teratogenic exposure during pregnancy. This case report underscores the importance of early recognition and differentiation between isolated and syndromic forms of congenital nail anomalies and highlights the favorable prognosis of isolated presentations. Parental counseling should emphasize the cosmetic nature of the condition, which may draw attention during school-age years and could potentially lead to psychosocial difficulties despite otherwise normal physical health.

## Case presentation

A full-term male neonate was delivered via spontaneous vaginal delivery to a 30-year-old primigravida mother with a history of gestational diabetes on a meal plan. To our knowledge, gestational diabetes has not been associated with congenital nail hypoplasia or anonychia, and no such link has been documented in the literature to date. The prenatal period was uneventful, with no maternal exposure to teratogenic drugs or infections. All antenatal ultrasonography revealed no abnormalities.

**Figure 1:**
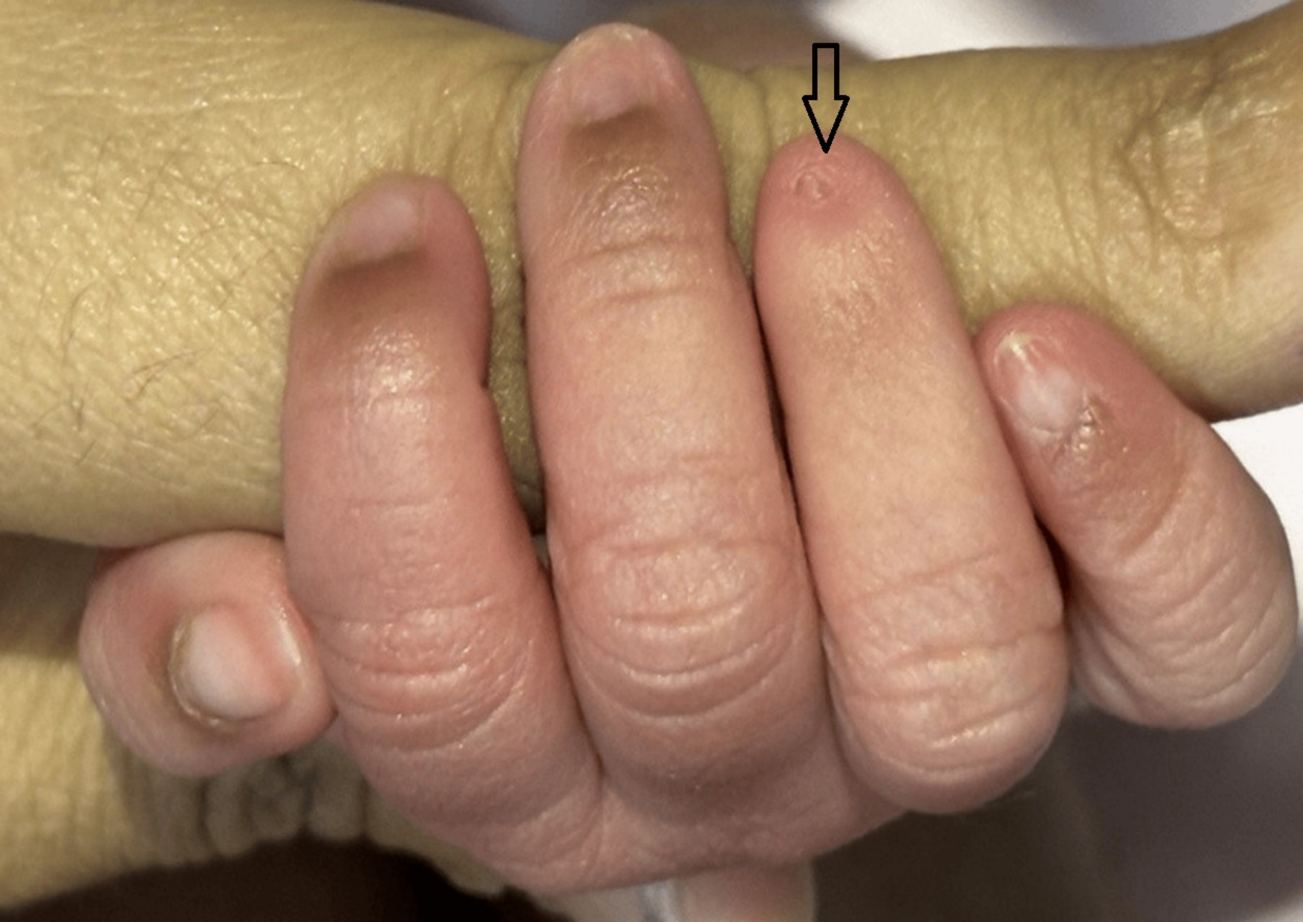
Right hand showing a hypoplastic nail on the ring finger (arrow)

**Figure 2:**
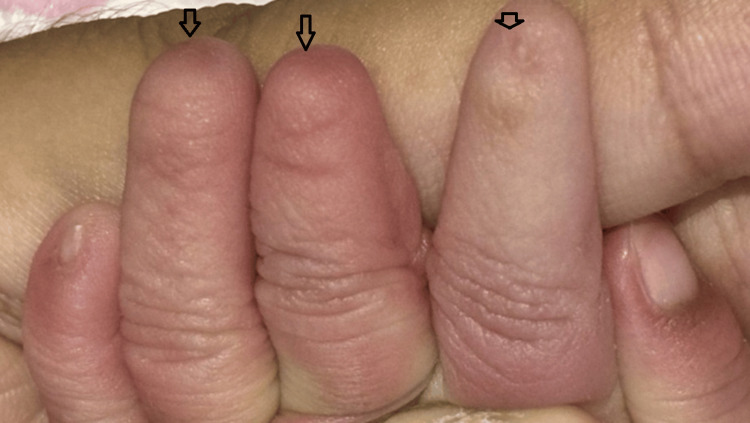
Left hand showing complete anonychia of the middle and ring fingers with a hypoplastic nail on the index finger (arrows)

**Figure 3:**
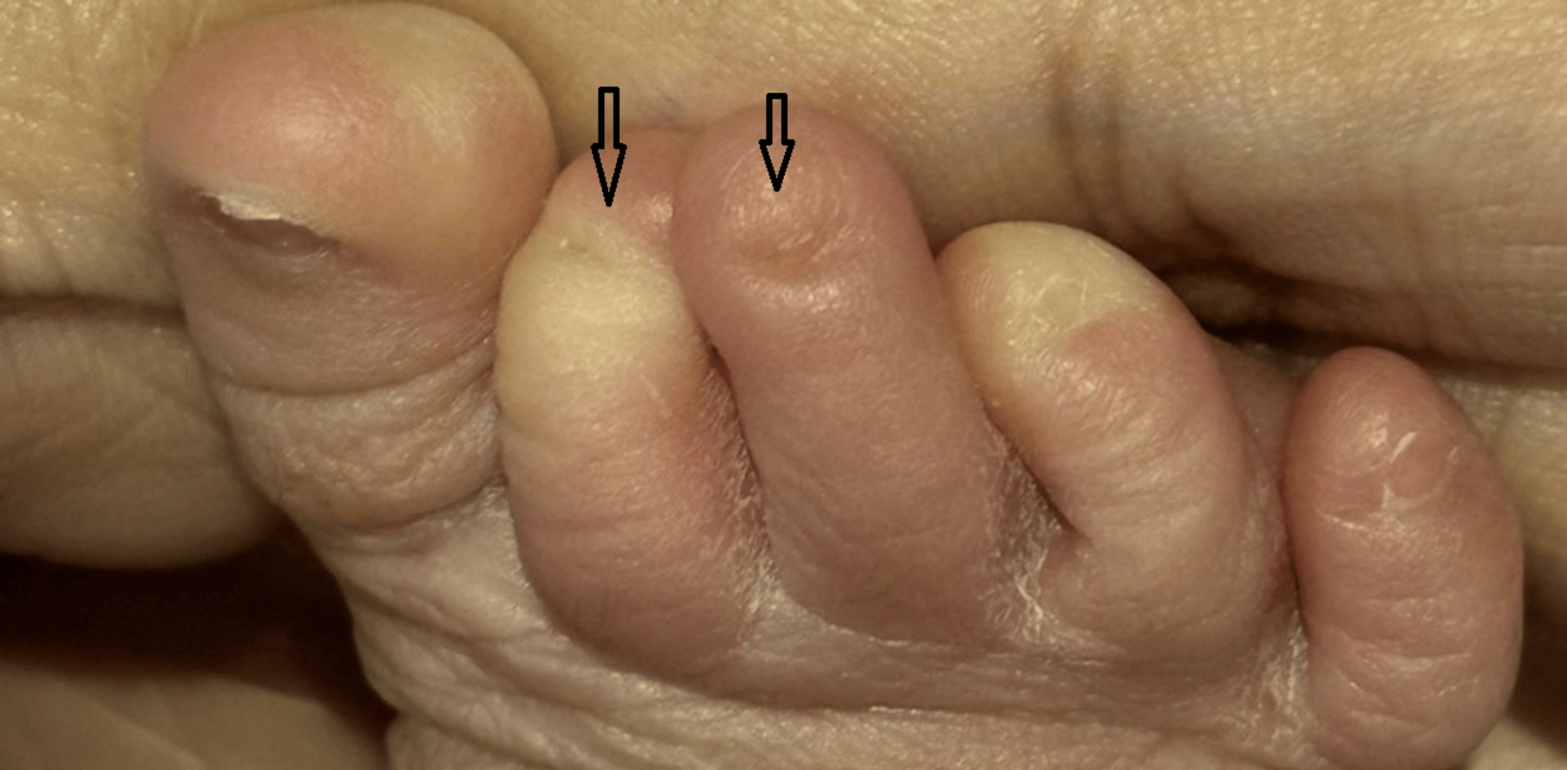
Right foot showing hypoplastic nails on the second and third toes (arrow)

**Figure 4:**
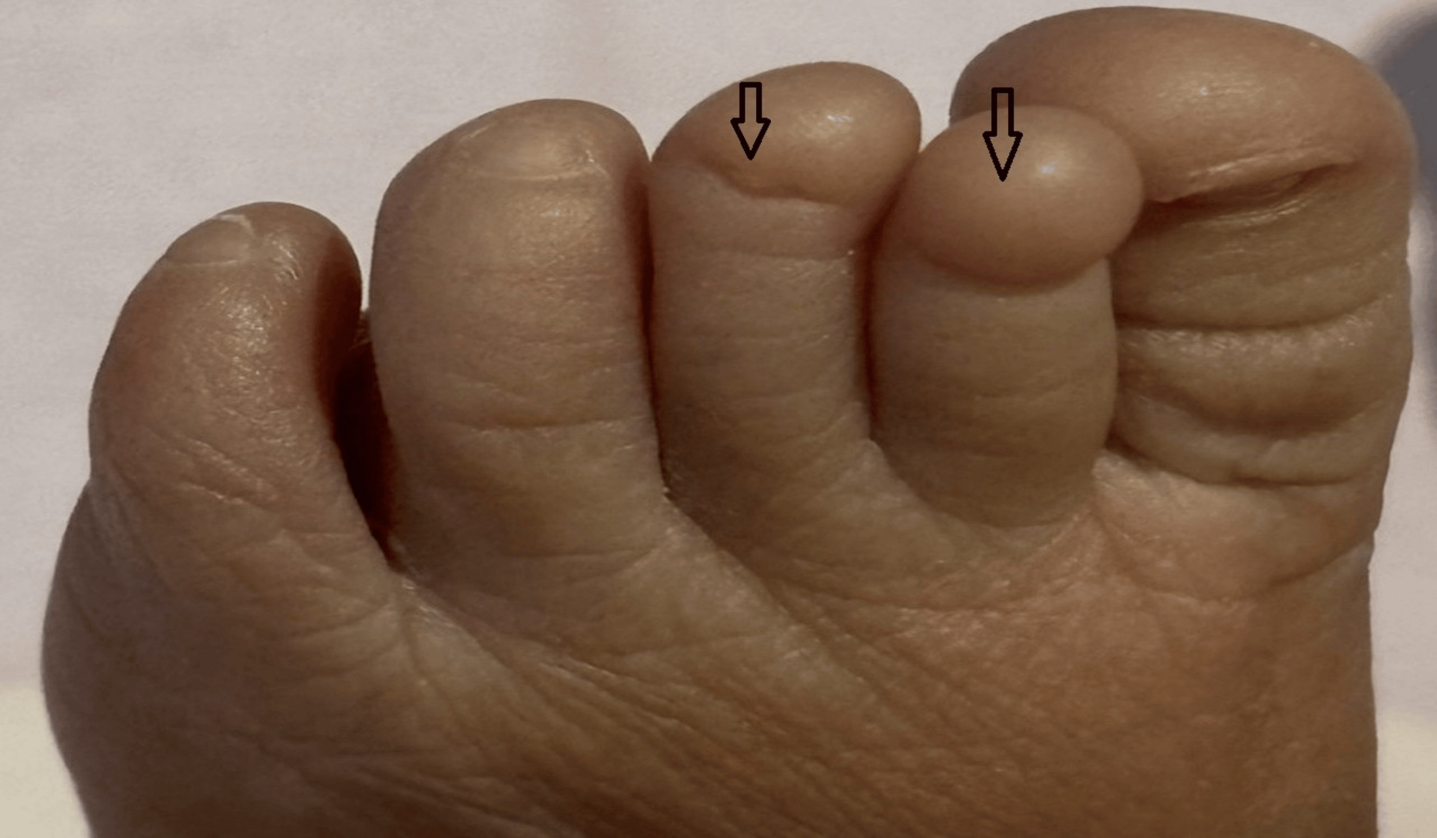
Left foot showing anonychia of the second toe and a hypoplastic nail on the third toe (arrow)

**Figure 5:**
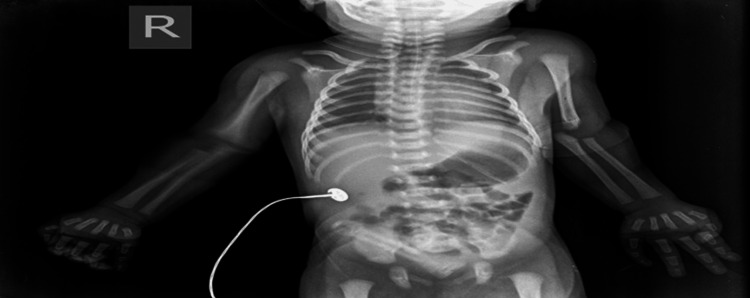
Radiograph of the upper limbs revealing normal morphology of the distal phalanges with no evidence of hypoplasia or aplasia

**Figure 6:**
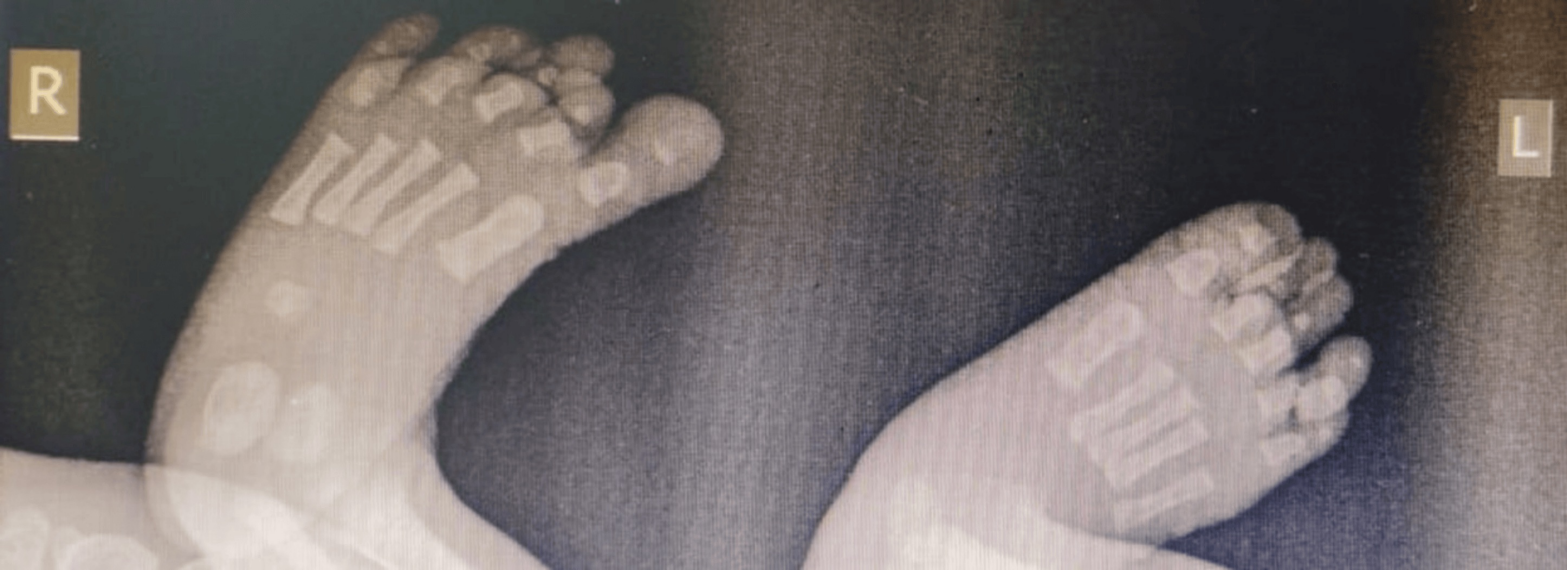
Radiograph of the feet showing the presence of all distal phalanges confirming the absence of associated bony defects

The neonate was delivered at 38 weeks of gestation with a birth weight of 2,560 grams. During the postnatal examination, soon after delivery, the newborn was noted to have partial and complete nail absence involving multiple digits on both upper and lower limbs. A detailed inspection of the limbs revealed a combination of both nail hypoplasia and anonychia. Specifically, the right hand’s fourth finger had a hypoplastic (underdeveloped) nail (Figure 1). The left hand exhibited a complete absence of nails on the third and fourth fingers, while the second finger displayed a hypoplastic nail (Figure 2). Similarly, examination of the right foot showed hypoplastic nails on the second and third toes (Figure 3). On the left foot, the second toe lacked a nail entirely, and the third toe had a hypoplastic nail (Figure 4). Radiographic evaluation of the chest and all limbs confirmed normal bone development, with no evidence of missing or malformed distal phalanges (Figures 5-6). The nail beds appeared normal, with no signs of inflammation, trauma, or scarring. Skin/hair/mucosa had normal texture, and no rashes, alopecia, or mucosal lesions were noted. No craniofacial, skeletal, or other systemic anomalies were observed.

Given the constellation of nail findings, a differential diagnosis was considered, including hereditary nail hypoplasia syndromes (e.g., DOOR syndrome, EEC syndrome, or Ellis-van Creveld syndrome), anonychia congenita, and ectodermal dysplasia. These conditions were excluded based on the absence of skeletal abnormalities, orofacial anomalies, seizures, cardiac defects, polydactyly, developmental delay, and ectodermal involvement. Notably, DOOR syndrome and EEC syndrome typically present with multisystem involvement, while Ellis-van Creveld syndrome is characterized by polydactyly and congenital cardiac defects, neither of which was present in this case. The absence of dysmorphic features, normal growth parameters, and preserved developmental milestones further supported the diagnosis of isolated congenital nail hypoplasia and anonychia.

On examination of the respiratory system, air entry was equal on both lung fields with no added sounds. On cardiovascular examination, S1 and S2 were normal, with no murmur, and on abdominal examination, no organomegaly was noted. Similarly, the central nervous system showed alertness, responsiveness, and normal tone and reflexes. Family history was negative for nail disorders or genetic syndromes. Given the isolated nature of the findings and absence of dysmorphic features, a diagnosis of isolated congenital hyponychia and anonychia was made. The parents were reassured about the condition's benign course. No further investigations or interventions were initiated.

The neonate remained clinically stable throughout the postnatal period and tolerated breastfeeding well. Follow-up assessments were conducted monthly during the first three months and then at three-month intervals over the subsequent year: growth parameters and developmental milestones, including gross motor, fine motor, language, and social domains, remained age-appropriate. No functional impairment related to grasping or digital motion was observed. During monitoring, the hypoplastic nails showed no signs of regeneration. Parents were counselled that long-term outcomes are generally favorable with minimal functional limitation. However, cosmetic concerns may arise later in childhood and can be addressed with nail prosthetics or reconstructive options if desired.

This case highlights several clinically meaningful aspects, including digit-specific nail involvement, normal osseous development, the isolated nature of the defect, and preservation of developmental milestones. These features reinforce the importance of differentiating isolated congenital nail anomalies from syndromic causes to avoid unnecessary investigations and alleviate parental anxiety.

## Discussion

A comprehensive review of the literature shows that isolated congenital nail hypoplasia and anonychia in neonates are exceedingly rare, with only a small number of well-documented case reports globally and even fewer originating from developing countries. The first macroscopic structures observed in nail morphogenesis are the nail field bordered by nail grooves, which appear at approximately 10 weeks of gestation, with nail plate formation initiated by 14-16 weeks; disruptions during this period can lead to congenital nail hypoplasia or absence [7].

Anonychia is a rare condition that can be inherited in an autosomal recessive manner and is associated with mutations in the RSPO4 gene, located on chromosome 20p13. These mutations, such as frameshift, splice-site, and missense changes in exon 2, affect the production of R-spondin 4, a protein crucial for WNT signaling that plays a key role in nail morphogenesis [8-10]. Although the genetic basis is well established, we did not pursue genetic testing due to financial constraints. Our case had no syndromic features, no family history, a normal skeletal examination, and the condition was clinically benign; in such cases, confirmatory testing would not alter management or prognosis. In some cases, anonychia may be acquired as a result of trauma, Stevens-Johnson syndrome, lichen planus, or epidermolysis bullosa [11,12]. Congenital anonychia can be associated with hypoplastic or missing underlying phalanges, microcephaly, curved digits, a single transverse palmar crease, bizarre flexural pigmentation, and hair abnormalities [13]. In contrast, our baby did not have any of these abnormalities.

Maternal use of certain medications, such as carbamazepine, phenytoin, warfarin, morphine, and trimethadione, particularly during the first and second trimesters, has also been implicated in anonychia. However, this factor did not pertain to our case. When isolated, these nail disorders are considered benign and do not affect physical development or quality of life. No specific treatment is required, and parental reassurance is the cornerstone of management. In this case, a thorough clinical evaluation ruled out associated anomalies, allowing the diagnosis of an isolated anomaly and avoiding unnecessary investigations. Follow-up is supportive and may be conducted through routine primary care, pediatric, or dermatology visits, with attention to psychological well-being during school-age years. Referral for psychological support can be considered if body image concerns or bullying arise. No specific therapeutic interventions are currently indicated for isolated presentations.

The lack of functional impairment associated with nail involvement may contribute to underreporting, especially in developing countries. Hence, we report a case exhibiting isolated nail anomalies in the absence of syndromic manifestations, consistent with a diagnosis of isolated congenital nail hypoplasia.

## Conclusions

Isolated congenital nail hypoplasia and anonychia are rare, benign conditions with an excellent prognosis. Accurate clinical examination with radiological confirmation of intact distal phalanges, as demonstrated in this case, is vital to distinguish isolated cases from syndromic presentations, thereby preventing over-investigation and allowing for reassurance of the family regarding the excellent prognosis. Structured follow-up is recommended to monitor developmental and psychosocial adaptation, with particular attention during periods of increased peer interaction. Counselling should include anticipatory guidance for parents, strategies to mitigate stigma (e.g., fostering positive body image, addressing bullying concerns), and discussion of cosmetic options if desired in adolescence. Increased awareness and proactive support can optimize long-term quality of life for affected children. A limitation of our case report is that we were unable to perform a genetic evaluation due to financial constraints.
